# Human Papillomavirus (HPV)-Positive Perianal Squamous Cell Carcinoma in a Patient With Hidradenitis Suppurativa and Psoriasis

**DOI:** 10.7759/cureus.77059

**Published:** 2025-01-07

**Authors:** Tatiana Hurtová, Monika Pecova, Katarina Simekova, Martin Jozef Pec, Lukas Plank, Karolina Vorcakova

**Affiliations:** 1 Department of Infectology and Travel Medicine, Jessenius Faculty of Medicine in Martin, Comenius University in Bratislava, Martin, SVK; 2 Department of Dermatovenerology, Jessenius Faculty of Medicine in Martin, Comenius University in Bratislava, Martin, SVK; 3 Department of Hematology and Transfusiology, Jessenius Faculty of Medicine in Martin, Comenius University in Bratislava, Martin, SVK; 4 Department of Internal Medicine I, Jessenius Faculty of Medicine in Martin, Comenius University in Bratislava, Martin, SVK; 5 Department of Pathological Anatomy, Jessenius Faculty of Medicine in Martin, Comenius University in Bratislava, Martin, SVK

**Keywords:** cancer screening, hidradenitis suppurativa, hpv, psoriasis, skin tumor, squamous cell carcinoma

## Abstract

Hidradenitis suppurativa (HS) is a chronic inflammatory skin disease that can lead to rare but aggressive complications, including squamous cell carcinoma (SCC), particularly in the anogenital region. Risk factors such as human papillomavirus (HPV) infection, chronic inflammation, and coexisting conditions such as psoriasis can increase the likelihood of malignant transformation in HS lesions.

We report a 43-year-old man with a history of HS (Hurley stage II) and psoriasis who developed a rapidly progressing perianal SCC. The patient, a chronic smoker, tested positive for low-risk HPV type 6 and high-risk HPV type 16. Imaging revealed an extensive tumor mass infiltrating the sacral bone and surrounding tissues. Histopathology confirmed well-differentiated SCC with inguinal lymph node metastasis. The patient underwent radical surgery and radiotherapy. Laboratory findings showed monoclonal immunoglobulin G (IgG) gammopathy and secondary thrombocytosis related to chronic inflammation.

SCC is a rare but serious complication of HS, particularly in the presence of chronic inflammation and HPV infection. Early detection through regular biopsies and vigilant monitoring of high-risk patients is essential. The consideration of HPV vaccination in severe HS cases, especially those undergoing immunosuppressive therapy, may reduce the risk of malignant transformation.

## Introduction

Hidradenitis suppurativa (HS) is a chronic inflammatory skin disease primarily affecting areas with apocrine glands: the axillae, groin, and perineum [1]. Painful nodules, abscesses, and scar creation characterize HS. Similarly, psoriasis is a chronic, immune-mediated skin condition that leads to the formation of erythematous, scaly plaques, predominantly affecting the extensor surfaces of the body [1]. The prevalence of HS has been reported to range from 1% to 4% of the general population, while that of psoriasis is estimated to be between 2% and 3% [1,2].

Squamous cell carcinoma (SCC) is the most severe but rare complication of HS [3]. SCC connected to HS is often very aggressive, with poor outcomes based on the late diagnosis of the SCC. Factors such as chronic inflammation, human papillomavirus (HPV) infection, and treatment-related immunosuppression can amplify the association between SCC and HS [4]. In our case, we present a 43-year-old man with a history of HS and psoriasis who developed aggressive SCC of the perianal region. This case underscores the importance of vigilance in monitoring HS patients for malignant transformations, particularly in the presence of comorbid conditions such as HPV infection.

## Case presentation

A 43-year-old Caucasian man, a chronic smoker (20 cigarettes/day), with a history of mild chronic plaque psoriasis (absolute Psoriatic Area and Severity Index under 3) and a body mass index (BMI) of 23.5 kg/m^2^, and an abstaining alcohol abuser and a casus socialis, presented at the department of dermatovenereology.

Six years prior to the first consultation, painful subcutaneous nodules in the axillary region and fistulas, nodules, and subcutaneous abscesses in the gluteal region developed, discharging a hemorrhagic-purulent content, classified as HS, repeatedly treated with systemic antibiotics and surgical incisions. At the same time, the patient was treated for small genitoanal condylomata acuminata with a combination of podophyllotoxin topically and cryosurgery. During an abuse recovery program and the reactive depressive syndrome treatment at the psychiatric department, the patient’s general practitioner observed the rapid progression of the perianal tumor.

**Figure 1:**
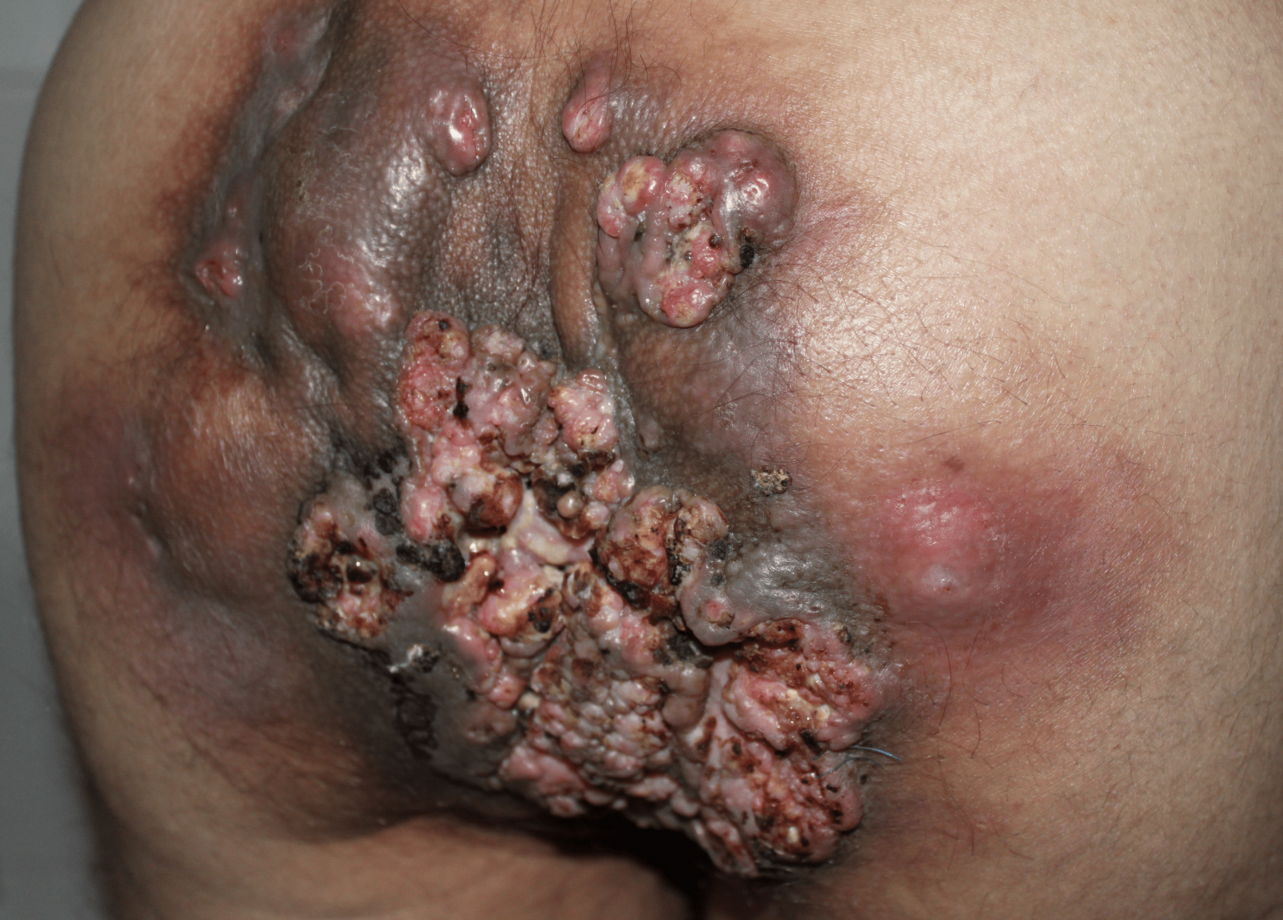
Verrucous squamous cell carcinoma of the gluteal region

**Figure 2:**
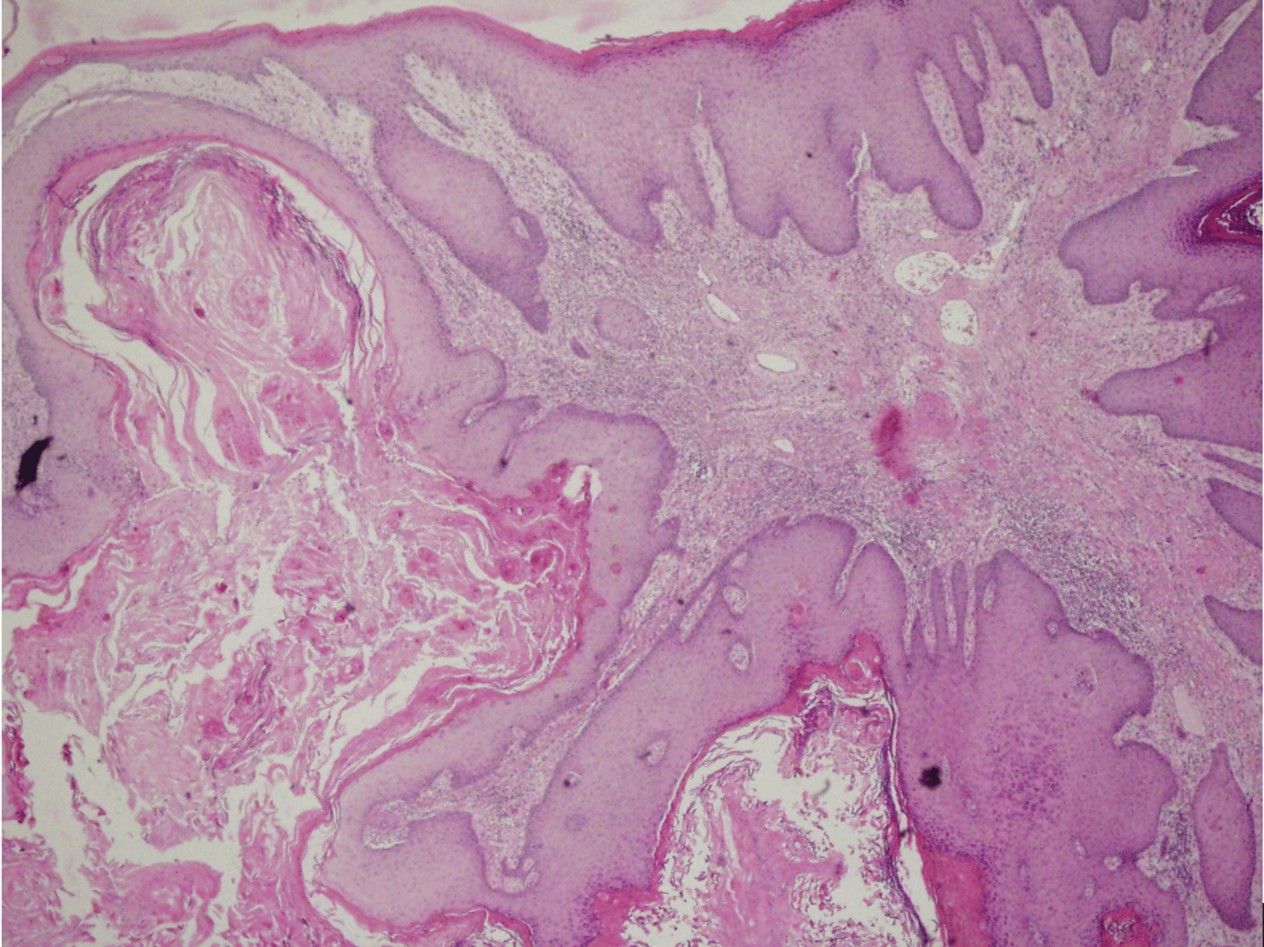
Verrucous exo- and endophytic growth of the epithelial tumor with expansive, demarcated growth and minimum cellular atypia In the basal layer of the epidermis, there is an increased mitotic activity and the occasional occurrence of atypical mitotic figures. There are intraepithelial infiltrates consisting of polymorphonuclear cells forming intraepidermal abscesses. The dermis is involved in a chronic inflammatory infiltration with recent and older hemorrhagic lesions (H&E, magnification: 120×)

At the first visit, the patient presented psoriasis of the knees, elbows, and scalp and gluteal tumor (Figure 1). Computed tomography verified an extensive gluteal mass with the destruction of the sacral bone, infiltrating the subcutaneous tissue, gluteal muscles, and rectum. From the tumor mass by the cultivation were isolated *Klebsiella pneumoniae*, *Proteus mirabilis*, *Alcaligenes odorans*, and *Bacteroides fragilis*. The biopsy from the tumor and inguinal lymph node histologically confirmed well-differentiated SCC (Figure 2) metastasizing to the inguinal lymph nodes with HPV type 6 and 16 positivity confirmed by real-time polymerase chain reaction (PCR).

In the axillary region, HS manifested as open double comedones and scars. With regard to the clinical picture in the genitoanal region before SCC development, the lesions were classified as HS Hurley stage II. Serum biochemistry revealed the reference values of minerals, urea, creatinine, uric acid, glycemia, bilirubin, liver enzymes, lactate dehydrogenase, lipids, ferritin, vitamin B12, homocysteine, immunoglobulin (Ig) A, IgM, and kappa/lambda ratio. The complete peripheral blood cell count confirmed no changes in erythrocytes, eosinophils, monocytes, or basophils. Coagulation, urine paraprotein, a prostatic cancer antigen, QuantiFERON-TB Gold test, treponema pallidum hemagglutination (TPHA), HIV p24Ag, hepatitis B and C viruses, and *Cytomegalovirus* were all negative.

The hematologist diagnosed a monoclonal IgG gammopathy, sideropenic anemia, secondary thrombocytosis due to chronic inflammation (Table 1), and an elevated erythrocyte sedimentation rate (106/hour).

**Table 1:** Pathological biochemical parameters at admission CRP, C-reactive protein; IgG, immunoglobulin G; HCT, hematocrit test; MCV, mean corpuscular volume; MCH, mean corpuscular hemoglobin; RDW, red cell distribution width; CEA, carcinoembryonic antigen; SCCA, squamous cell carcinoma antigen; ELFO, electrophoresis

Parameters	Values	Reference values	Units
CRP	97.5	0.0-5.0	mg/L
IgG	23.5	7.0-16.0	g/L
Albumin	29.0	35.6-50.0	g/L
ELFO albumin	0.146	0.520-0.670	%
ELFO beta	0.187	0.090-0.150	%
ELFO gamma	0.578	0.120-0.220	%
Kappa light chains	65.34	3.30-19.40	mg/L
Lambda light chains	49.66	5.71-26.30	mg/L
Serum Fe	2.1	7.2-21.5	umol/L
Transferrin	1.84	2.00-3.60	g/L
Iron saturation	4.55	20.00-50.00	%
Leukocytes	16.0	3.90-10.00	10×9/L
Hemoglobin	84	140-179	g/L
HCT	0.29	0.39-0.54	L
MCV	66.7	82.0-98.0	fL
MCH	19.4	26.0-34.0	Pg
RDW	24.83	11.6-14.5	%
Platelets	700	140-400	10×9/L
Neutrophils	77.4	45.0-72.0	%
Lymphocytes	14.4	25.0-46.0	%
SCCA	7.6	0.0-1.5	ng/mL
CEA	4.81	0.0-3.4	ng/mL
Beta-2 microglobulin	2.54	0.8-1.8	mg/L
Paraprotein	18.8	0.0	G

The patient underwent axial resection of the sigmoid colon and treatment with intravenous antibiotics (cefuroxime, ciprofloxacin, and cefoperazone-sulbactam) and received numerous blood transfusions and iron supplements following radical surgical treatment and radiotherapy.

## Discussion

Squamous cell carcinoma is a rare but potentially fatal complication of HS. The most important risk factors of SCC are sun exposure, age, immunosuppression, and fair skin, but SCC was also described in chronic wounds, chronic inflammatory processes, or sclerosing conditions [4]. The first case of SCC and HS was described in 1958 by Anderson and Dockerty [5]. Since then, more studies have confirmed the association between HS and SCC, particularly in the perianal and gluteal regions of long-standing HS patients [6]. The pathogenesis of SCC in HS is different than SCC rising from sun-exposed areas. Fabbrocini et al. created the theory to explain the association between HS and SCC, an immunocompromised cutaneous district. This concept suggests that areas of chronically affected skin exhibit localized immune dysfunction, creating conditions that permit tumor or infection development [7]. Lymphatic stasis, a common complication in HS, exacerbates this effect by disrupting the normal flow of immune cells to the lymphedematous area, thereby increasing the risk of malignancy [8]. Additionally, the stroma in a chronic, nonhealing wound, which is rich in growth factors such as platelet-derived growth factor (PDGF) and vascular endothelial growth factor (VEGF), further promotes tumor growth in these regions [9].

The risk factors of SCC in HS are male gender, long disease duration (often more than 20 years), smoking, chronic inflammation, and HPV infection. Racanelli et al. have done a systematic review and individualized patient data meta-analysis of 138 patients with HS and cutaneous SCC [4]. Their study revealed that the mean lead time from the onset of HS to SCC was 24.7 years. The majority of the patients had severe HS (Hurley stage III), with the most affected site being gluteal/perianal. Diagnosing SCC in HS lesions is difficult; therefore, a repeated biopsy by deep excision is recommended. Postoperative actinotherapy reduces disease recurrence by 30% [10].

HPV was detected in more than 30% of patients with HS and SCC [4]. High association with HPV can be an explanation for why the SCC in HS occurs in the gluteal area. HPV is known to be an important part of the pathogenesis of skin and mucosal SCC because HPV prevents apoptosis and allows viral DNA replication [11]. Lavogiez et al., using highly sensitive PCR methods, confirmed the presence of low-risk alpha HPV (HPV type 6) in all tissue samples of eight SCC patients and high-risk alpha HPV (HPV types 16 and 68) and beta-HPV (types 17, 22, 23, 38, 76, and 80) [12]. The investigations for HPV infection and HPV vaccination may be considered before the administration of anti-tumor necrosis factor (TNF)-alpha agents. The one- and five-year survival rates of patients with HS and SCC are 63.4% and 38.5%, respectively [4]. The most important factors that contribute to poor outcomes are ulcerative morphology, clinical stage, and higher histologic grade of SCC. In the future, it will be necessary to consider therapeutic approaches to improve the outcomes of patients with HS and SCC.

In contrast to our patient, singularly, it is relatively short from disease onset (six years) and has a lower Hurley stage of HS (stage II). The coincidence of psoriasis in our patient is very unusual. To our knowledge, this is the first case of a patient with HS, SCC, and psoriasis. HS and psoriasis are both chronic inflammatory skin diseases. A large-scale population-based study revealed a significant association between HS and psoriasis. Patients with psoriasis have an 80% increase in the odds of HS when compared to healthy controls [13] and vice versa; psoriasis was significantly increased in patients with HS compared to the control group (OR: 4.6) [14]. The link between these two conditions should be interleukin-12, interleukin-23, and tumor necrosis factor-alpha [15]. A study by Asgari et al. showed an increased incidence of SCC among patients with systemically treated psoriasis with exposition to biologics (majority of TNF-alpha inhibitors) [16]. Chronic systemic inflammation and immunosuppressive medications are considered to be risk factors for cancer in patients with psoriasis [17]. The coincidence of psoriasis, HS, and SCC is very rare; it is necessary to add more robust data if the chronic systemic inflammation, multiplied by psoriasis and HS, can shorten the period from the onset of HS to the development of SCC.

Monoclonal gammopathy of undetermined significance (MGUS) is used to describe the presence of a clone of plasmocyte cells in the bone marrow without signs of malignant proliferation or damage to the organism by monoclonal immunoglobulin or the clonal proliferation of the plasmocytes. MGUS is usually a predecessor to multiple myeloma (MM), with an average annual 1% risk of progression to MM or other lymphoproliferative disease [18]. Autoimmune disease may play a role in the development of MGUS and myeloma, as chronic inflammation is associated with cancer, including lymphoproliferative diseases [19]. Recently, a comprehensive literature review hinted at the increased prevalence of autoimmune disorders in patients with MM and MGUS, including pernicious anemia, autoimmune hemolytic anemia (AIHA), pure red cell aplasia, immune thrombocytopenia, autoimmune neutropenia, rheumatoid arthritis, systemic lupus erythematosus, dermatomyositis and polymyositis, Sjogren’s syndrome, ankylosing spondylitis, leukocytoclastic vasculitis, autoimmune neurologic disorders, and irritable bowel disease (IBD) [20].

## Conclusions

The case presented here involves the rare fast progression (two years) of HS Hurley stage II lesions into well-differentiated SCC with HPV type 6 and 16 positivity. Chronic severe inflammation resulted in monoclonal IgG gammopathy with positive paraprotein and kappa and lambda chains, sideropenic anemia, and secondary thrombocytosis. A low five-year survival rate emphasizes the importance of early detection and regular examination of patients with HS to improve prognosis. In the future, more research is necessary to screen SCC lesions in HS patients. The presence of high-risk HPV may be a significant risk factor for the malignant transformation of lesions and, therefore, should be investigated in patients with severe HS lesions. Another question is the role of HPV vaccination in high-risk patients in the prevention of SCC in patients with HS.
